# The discrimination of facial sex in developmental prosopagnosia

**DOI:** 10.1038/s41598-019-55569-x

**Published:** 2019-12-13

**Authors:** Jade E. Marsh, Federica Biotti, Richard Cook, Katie L. H. Gray

**Affiliations:** 1 School of Psychology and Clinical Language Sciences, University of Reading, Reading, UK; 2 Department of Psychology, Royal Holloway, University of London, Egham, UK; 3 Department of Psychological Sciences, Birkbeck, University of London, London, UK

**Keywords:** Human behaviour, Sensory processing

## Abstract

Developmental prosopagnosia (DP) is a neurodevelopmental condition characterised by difficulties recognising and discriminating faces. It is currently unclear whether the perceptual impairments seen in DP are restricted to identity information, or also affect the perception of other facial characteristics. To address this question, we compared the performance of 17 DPs and matched controls on two sensitive sex categorisation tasks. First, in a morph categorisation task, participants made binary decisions about faces drawn from a morph continuum that blended incrementally an average male face and an average female face. We found that judgement precision was significantly lower in the DPs than in the typical controls. Second, we used a sex discrimination task, where female or male facial identities were blended with an androgynous average face. We manipulated the relative weighting of each facial identity and the androgynous average to create four levels of signal strength. We found that DPs were significantly less sensitive than controls at each level of difficulty. Together, these results suggest that the visual processing difficulties in DP extend beyond the extraction of facial identity and affects the extraction of other facial characteristics. Deficits of facial sex categorisation accord with an apperceptive characterisation of DP.

## Introduction

Developmental prosopagnosia (DP) is a neurodevelopmental condition characterised by difficulties recognising and discriminating faces^1–3^. Individuals with DP may learn to distinguish others based on non-facial features, such as hair, clothing, voice, or gait^4^. DP is evident in the absence of manifest brain injury and other psychiatric disorders (e.g. autism, schizophrenia), although co-occurring deficits in object processing are not uncommon^5–7^. The face processing impairments seen in DP appear to affect the perception of both same- and other-ethnicity faces^8^. Current estimates suggest that ~2% of the general population may experience lifelong face recognition difficulties severe enough to disrupt their daily lives^9,10^. Consistent with other neurodevelopmental disorders, DP often runs in families, indicating that the condition may have a genetic component^11–13^.

The nature of the impairment in DP remains unclear. Mnemonic accounts propose that DPs form accurate structural descriptions of faces, but cannot maintain these representations over time^14–19^. Proponents of this view point to the fact that some DPs score worse on standardised tests of face perception with a mnemonic component, than on standardised tests with little or no memory demands^15,18,20^. Other researchers favour an apperceptive account – that DP is an impairment of early structural face-encoding^1,3,21,22^. Consistent with this view, the relative size of face matching deficits seen in DP do not vary as a function of retention interval^23,24^ and many DPs struggle to sort unfamiliar faces presented simultaneously^4,23^.

A prediction of the apperceptive account is that the perceptual impairments found in DP ought to extend beyond identity processing. Leading models of face processing propose that a common structural description, formed early in the face processing stream, informs subsequent processing of identity, expression, age, and sex, that occurs in largely divergent pathways^25,26^. If the problems seen in DP arise from noisy structural descriptions, impairments should be seen, not only when judging identity, but also when judging the expression, age, and sex of faces. Nevertheless, whether or not the perceptual deficits seen in DP extend beyond identity processing remains controversial.

The question that has attracted most research attention is whether DPs have deficits of facial expression processing. Although case-studies have described individual DPs with expression recognition deficits^27,28^, early group studies failed to detect differences between DPs and controls^29–31^. However, many of the tasks used in the early group studies lacked sensitivity; for example, when asked to categorise prototypical ‘basic emotions’ the performance of typical observers approaches ceiling. Having used image morphing to render facial expression stimuli more ambiguous, Biotti and Cook^32^ found that a sample of DPs (*N* = 15) showed significantly poorer facial emotion categorisation precision than matched controls.

The present study sought to determine whether the perceptual problems seen in DP extend to another non-identity attribute - judgements about facial sex. Here again, the existing experimental findings on sex recognition in DP paint an inconsistent picture. Studies of individual cases suggest that discrimination of facial sex may be impaired in some cases of DP^2,33–36^, but not in others^29,37–39^. Group studies have also yielded equivocal results; some authors have described similar levels of performance in DPs and typical observers^29,40–42^, while others have described weak differences at the group level^43^.

The present study investigated facial sex discrimination in a group of DPs using sensitive psychophysical tasks. In both tasks, external cues to sex were eliminated; participants were forced to use the internal structure of a briefly presented face to make binary ‘male’/’female’ categorisation decisions. In a morph categorisation task, participants made binary decisions about faces drawn from a morph continuum that blended incrementally an average male face and an average female face. In a sex discrimination task, participants made binary decisions about female or male facial identities that had been blended with an androgynous average face. To vary task difficulty, we manipulated the relative weighting of each facial identity and the androgynous average to create four levels of signal strength. Relative to typical controls, we found that DPs were i) less precise in their categorisations of facial sex (morph categorisation task), and ii) less sensitive to the sex signal presented in faces at all levels of task difficulty (sex discrimination task).

## Methods

### Diagnostic testing

Seventeen adults with DP (7 males; *M*_age_ = 45.9 years, *SD*_age_ = 11.8 years) were recruited through www.troublewithfaces.org. All reported life-long face recognition difficulties in the absence of brain injury or psychiatric disorder (e.g. autism, schizophrenia). Diagnostic decisions were based on participants’ scores on the Twenty-Item Prosopagnosia Index (PI20)^44,45^ and the Cambridge Face Memory Test (CFMT)^22^. We also report the DPs’ performance on the Cambridge Face Perception Test (CFPT)^11^ to index their face encoding ability, and the Cambridge Car Memory Test (CCMT)^46^, a measure of non-face object recognition ability. Diagnostic information for each DP is provided in Table 1. Ethical approval was granted by the University of Reading’s ethics committee. The study was conducted in line with the ethical guidelines provided by the 6th (2008) Declaration of Helsinki. All participants provided informed consent and were fully debriefed after the experimental procedure.

**Table 1:** Scores for each developmental prosopagnosic (DP) on the Twenty-Item Prosopagnosia Index (PI20), the Cambridge Face Memory Test (CFMT), the Cambridge Face Perception Test (CFPT), and the Cambridge Car Memory Test (CCMT), with z-scores in parentheses.

Participant	Age	PI20	CFMT [%]	CFPT [Errors]	CCMT [%]
F1	56	88 (−5.50)	47.22 (−4.23)	64 (−3.70)	76.39 (−0.23)
F2	31	72 (−3.74)	51.39 (−3.77)	60 (−3.27)	50 (−1.87)
F3	35	86 (−5.28)	45.83 (−4.39)	58 (−3.06)	69.44 (−0.32)
F4	36	78 (−4.40)	56.94 (−3.14)	50 (−2.20)	65.28 (−0.66)
F5	22	70 (−3.52)	59.72 (−2.83)	—	—
F6	40	90 (−5.72)	50.00 (−3.92)	66 (−3.91)	55.56 (−1.43)
F7	38	78 (−4.41)	63.89 (−2.36)	40 (−1.13)	56.94 (−1.32)
F8	68	79 (−4.51)	61.11 (−2.67)	40 (−1.13)	66.67 (−0.54)
F9	44	76 (−4.18)	59.72 (−2.83)	52 (−2.42)	56.94 (−1.32)
F10	60	82 (−4.84)	62.28 (−2.54)	42 (−1.35)	79.17 (0.45)
M1	48	77 (−4.29)	54.17 (−3.45)	60 (−3.27)	66.67 (−0.54)
M2	58	86 (−5.28)	58.33 (−2.99)	42 (−1.35)	87.5 (1.11)
M3	49	76 (−4.18)	54.17 (−3.45)	76 (−4.98)	83.33 (0.78)
M4	57	86 (−5.28)	54.17 (−3.45)	38 (−0.92)	81.94 (0.67)
M5	41	79 (−4.51)	45.83 (−4.39)	34 (−0.49)	55.56 (−1.43)
M6	50	79 (−4.51)	56.94 (−3.14)	42 (−1.35)	65.28 (−0.66)
M7	47	83 (−4.95)	52.78 (−3.61)	60 (−3.27)	52.78 (−1.65)
DP mean	45.9	80.29	54.97	51.5	66.84
DP SD	11.8	5.58	5.3	11.51	11.49
Comparison Mean	29.23	37.96	84.98	29.41	73.52
Comparison SD	11.91	9.09	8.92	9.35	12.57

Note: To calculate z-scores, DPs’ scores on each diagnostic test were compared to a sample of typically developed controls who completed the tests under lab conditions: PI20, CFMT, and CFPT comparison sample of 54 (23 males) controls^23^ (Experiment 2); CCMT, comparison sample of 61 (27 males) controls^6^.

### Morph categorisation task

**Figure 1:**
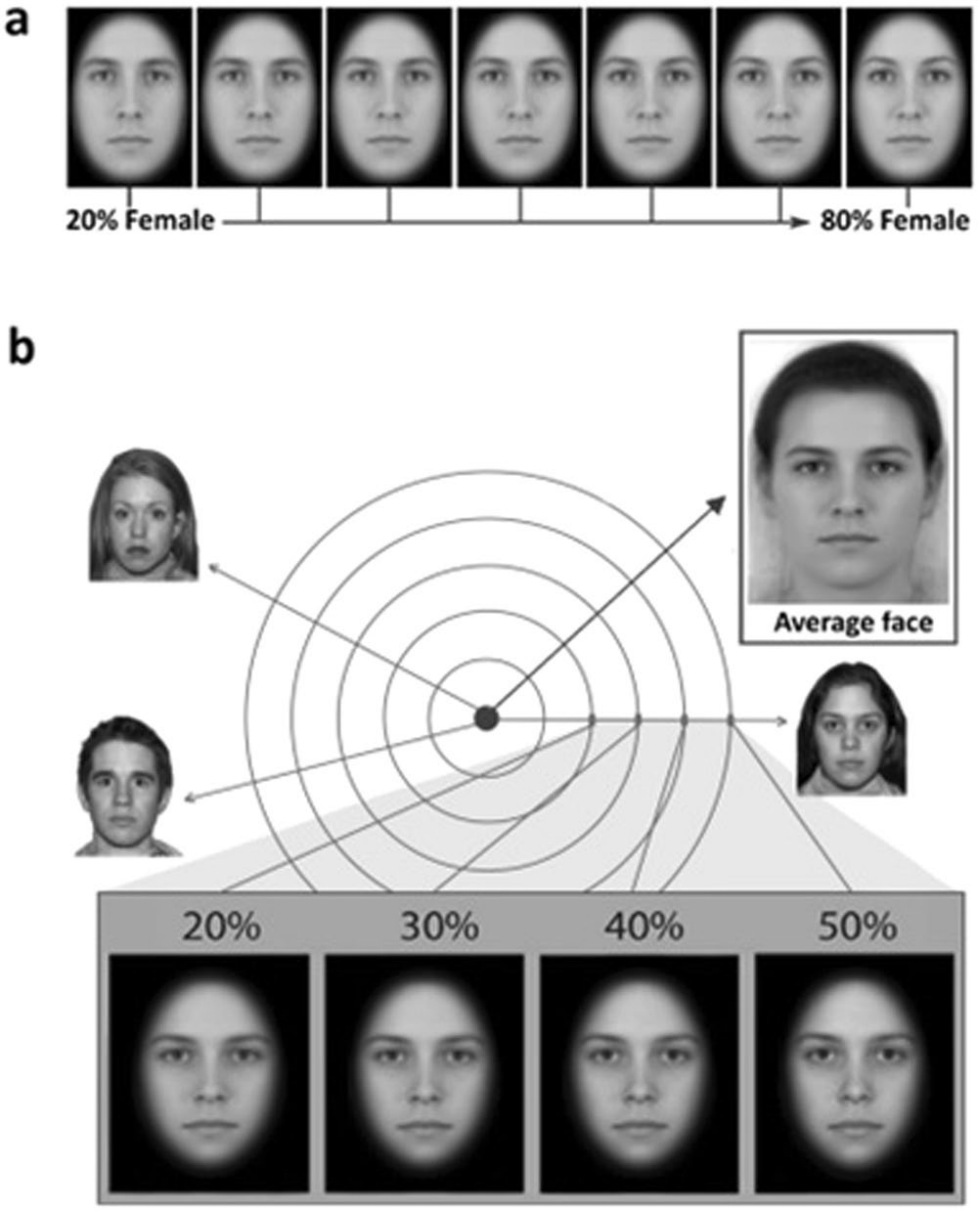
(**a**) Morphed stimuli used in the morph categorisation task. One male and female model were morphed along a continuum, ranging from 20% (80% male) to 80% female (20% male) in 10% increments. (**b**) Stimuli used in the sex discrimination task. Forty models were morphed together to create an androgynous average face. Each model was then morphed with the androgynous face to create four weighted morphs (20%, 30%, 40% and 50% identity/sex signal) along a continuum for each model.

In this task, a central fixation cross (250 ms) was followed by a single face presented centrally (250 ms), followed by a high-contrast mask (250 ms). Each stimulus image was drawn from a morph-continuum that blended an average male face with an average female. Facial-sex cues ranged in intensity from 20% female to 80% female in 10% increments (see Fig. 1a). Participants were asked to categorise the stimulus image presented as either male or female. We used participants’ responses to model psychometric functions that plotted how their response likelihoods varied as a function of the sex signal contained in the face.

Stimulus images were presented in greyscale and subtended 7.9° × 5.9° of visual angle when viewed at a distance of 60 cm. Hair and jaw lines were occluded by an elliptical window with a Gaussian blur at the edges. Each of the seven morphs were presented 20 times, in a random order, resulting in 140 trials.

The average male face and average female face used to create the morph continuum were themselves created by morphing together 16 males and 16 females, respectively. The original images were downloaded from the Chicago Face Database (CFD)^47^, and morphed using the method described by Adams, Gray, Garner and Graf^48^.

The performance of the DPs was compared with twenty typically developed (TD) controls recruited from the local subject pool (5 males; *M*_age_ = 41.2 years, *SD*_age_ = 8.3 years). As expected, the TDs (*M* = 41.1, *SD* = 10.4) had significantly lower PI20 scores than DPs [*t*(35) = 13.97, *p* < 0.001]. Neither participant age [*t*(35) = 1.43, *p* = 0.162] nor proportion of males [*X*^2^ (1) = 1.10, *p* = 0.295] differed significantly between the two groups.

### Sex discrimination task

A central fixation cross (250 ms) was followed by a single face presented centrally (250 ms) drawn from a set of 40 to-be-judged identities (20 male, 20 female). A high-contrast mask was presented for 250 ms immediately after stimulus offset. Participants were asked to categorise each face as male or female. We measured participants’ ability to discriminate male and female faces using signal detection theory^49^.

To make the task more challenging we diluted the sex signal in each stimulus image by morphing the facial identity with an androgynous average face^48^. The androgynous face was constructed by morphing together all 40 of the to-be-judged facial identities. To produce four levels of task difficulty, we varied the relative weighting of each facial identity and the androgynous average (Fig. 1b). In the most difficult condition, stimulus images contained only 20% of the original facial identity; in the least difficult condition, stimulus images contained 50% of the original facial identity.

The original facial images were sourced from the NimStim face set^50^ and the CFD^47^. Images were presented in greyscale and subtended 7.3° × 5.4° of visual angle when viewed at 60 cm. Each facial identity was presented four times at each of the morph levels in a random order resulting in 640 trials (40 facial identities × 4 morph levels × 4 repetitions). Both experiments were programmed in MATLAB, using the Psychophysics Toolbox extensions^51,52^.

The performance of the DPs was compared with twenty TD controls recruited from the local subject pool (5 males; *M*_age_ = 43.5 years, *SD*_age_ = 7.5 years). Twelve out of twenty TDs from this sample also completed the sex categorisation task. Neither participant age [*t*(35) = .76, *p* = .452] nor proportion of males [*X*^2^ (1) = 1.10, *p* = 0.295] differed significantly between the two groups. Once again, the TDs (*M* = 41.1, *SD* = 11.0) had significantly lower PI20 scores than DPs [*t*(35) = 13.34, *p* < 0.001].

## Results

### Morph categorisation task

We used each participants’ binary choice responses to estimate their psychometric function by fitting Cumulative Gaussian functions using the Palamedes toolbox^53^. We were primarily interested in the slope of participants’ psychometric function, quantified using the reciprocal of the standard deviation of the symmetric Gaussian distribution underlying each cumulative Gaussian function. Higher slope values (steeper slopes) indicate that observers can perceive subtle differences between the stimuli and vary their responses accordingly. Slope values and the point of subjective equivalence (PSE) for DPs and TD controls were compared using independent samples *t*-tests (two-tailed) (α = 0.05).

**Figure 2:**
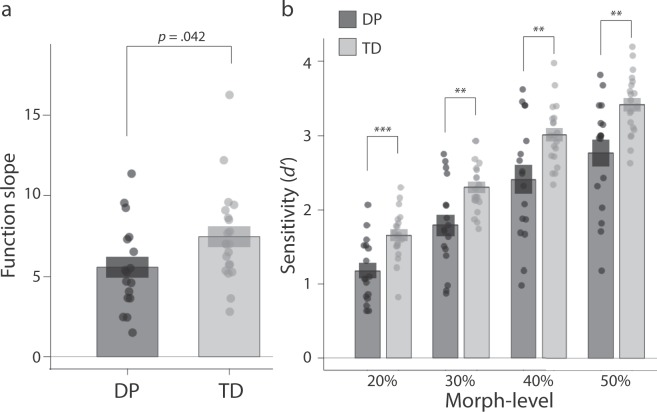
Results from (**a**) the morph categorisation task and (**b**) the sex discrimination task. Note: bar height gives the mean, bands give the mean ± 1 SEM; *** denotes *p* < 0.001, ** denotes *p* < 0.01.

There was a significant difference between slope estimates for DPs (*M* = 5.86, *SD* = 2.86) and TDs (*M* = 7.97, *SD* = 3.14) [*t*(35) = 2.116, *p* = 0.042 (two-tailed)], indicating that at the group level, the DPs were less sensitive to subtle differences in facial sex than TD controls (see Fig. 2a). There was no difference in PSE between the DPs (*M* = 0.50, *SD* = 0.08) and TD controls (*M* = 0.53, *SD* = 0.05) [*t*(35) = 1.15, *p* = 0.258 (two-tailed)], suggesting that the facial sex categorisation boundary was similar for both groups.

While the groups did not differ significantly, the DP group contained a slightly higher proportion of male observers than the group of typical controls. To confirm that observer gender had no influence on our results, we re-ran the foregoing analyses in an ANOVA with observer gender as an additional between-subjects factor. None of the main effects or interactions with observer gender approached significance for either the slope (all *F*’s < 0.1, all *p*’s > 0.75) or PSE analyses (all *F*’s < 2.0, all *p*’s > 0.17).

### Sex discrimination task

For the purpose of the signal detection analysis, a female response to a female stimulus was regarded as a hit, whereas a female response to a male stimulus was regarded as a false alarm. We derived measures of discrimination sensitivity (*d*′ = z[hit rate] – z[false alarm rate]) and bias (*c* = −0.5(z[hit rate] + z[false alarm rate])) at each level of task difficulty. When hit or false alarm rates were 1 or 0 (respectively), total hits or false alarms were adjusted by 0.5 before calculating *d*′.

To assess sensitivity, *d’* values were submitted to ANOVA (α = 0.05) with Difficulty (20%, 30%, 40%, 50%) as a within-participants factor and Group (TD, DP) as a between-participants factor (Fig. 2b). Sphericity violations were corrected using the Greenhouse-Geisser adjustment. The analysis revealed a main effect of Difficulty [*F*(3, 105) = 219.06, *p* < 0.001, η_p_^2^ = 0.86], indicating that as the strength of the sex signal in the stimuli increased, participants’ sensitivity also increased. Importantly, there was also a main effect of Group [*F*(1, 35) = 14.16, *p* = 0.001, η_p_^2^ = 0.29], whereby DPs were less sensitive than TDs. DPs were less sensitive than TDs at each Difficulty level (all *p*s < 0.01). The effect of Group did not vary as a function of Difficulty [*F*(3, 105) = 0.65, *p* = 0.585, η_p_^2^ = 0.02].

To assess bias, *C* values were submitted to ANOVA with Difficulty (20%, 30%, 40%, 50%) as a within-participants factor and Group (TD, DP) as a between-participants factor. There was a significant main effect of Difficulty [*F*(1.76, 61.69) = 42.84, *p* < 0.001, η_p_^2^ = 0.55]. At low morph-levels, participants were slightly biased to respond ‘female’, whereas at higher morph-levels, participants were slightly biased to respond ‘male’ (see Supplementary Materials for more details). There was no main effect of Group [*F*(1, 35) = 2.18, *p* = 0.149, η_p_^2^ = 0.06], and no Group × Difficulty interaction [*F*(1.76, 61.69) = 0.24, *p* = 0.760, η_p_^2^ = 0.01].

Once again, we re-ran the foregoing analyses with observer gender as an additional between-subjects factor. None of the main effects or interactions with observer gender approached significance for either the sensitivity (all *F*’s < 1.60, all *p*’s > 0.19) or bias analyses (all *F*’s < 1.50, all *p*’s > 0.20). Z-scores for each DP on both tasks are presented on Table S1 in the Supplementary Materials.

## Discussion

The present study aimed to determine whether facial sex discrimination is impaired in DP. In our morph categorisation task, we found that judgment precision, inferred from the slope of individual psychometric functions, was significantly lower for DPs relative to controls. We replicated and extended this finding using a sex discrimination task. We found that DPs were significantly less sensitive to facial sex cues compared to controls at all levels of task difficulty. Despite their impairments, DPs did not differ significantly from controls in terms of categorisation threshold (PSE) or response bias (*C*).

Previous studies of facial sex categorisation in DP have provided equivocal results. Several studies have found no difference in task performance between DPs and typical controls^29,41,42^. To date, only one study has reported evidence of atypical perception of facial sex in DP at the group level^43^. In their study, Esins *et al*.^43^ compared 16 DPs and 21 controls, and whilst both groups were approaching ceiling (controls: 91.5% correct; DPs: 84.4% correct), DPs were significantly impaired when making binary classifications of facial sex.

We speculate that methodological issues may have prevented earlier studies from detecting the clear, widespread differences seen here. In previous studies, participants were asked to categorise the sex of unmodified facial images^29,41,42^. This may have let DPs use trivial pictorial cues, such as the presence of make-up or facial hair – and in some cases, actors’ hair line^29^ – to achieve broadly typical levels of categorisation accuracy. In several studies, stimuli were also held on screen until response execution^39,42^, thereby permitting DPs to detect pictorial cues and develop other heuristics. Our use of image-morphing to vary the strength of sexually dimorphic signals, together with brief presentation durations, ensured that our psychophysical tasks taxed the structural encoding processes thought to be aberrant in DP.

The widespread sex discrimination deficits seen here provide compelling support for the apperceptive account of DP. Our tasks had little or no memory component – participants had to make a simple binary-choice decision about a single stimulus. Unlike sequential matching or delayed match-to-sample tasks, there was no need to hold the target face in memory; rather, participants were free to determine their response at any point after stimulus onset. The impairments seen here are therefore hard to explain in terms of a mnemonic deficit. Instead, the fact that DPs were impaired on our sex categorisation tasks strongly suggests a problem forming structural descriptions of faces. Leading models of face processing propose that a common structural description, formed early in the face processing stream, informs subsequent processing of identity, expression, age, and sex, mediated by largely independent, divergent pathways^25,26^. Subtle deficits of facial sex, and facial emotion perception^33^, together with problems sorting simultaneously presented faces by similarity^24^, suggest that this early structural description may be impoverished in DP.

The nature of the apperceptive deficit seen in DP remains currently unclear. According to one influential account, a failure to process faces holistically – whereby facial features are integrated into a non-decomposable whole^54–56^ – may underlie the face recognition deficits seen in DP^31,57–60^. However, recent evidence suggests that most individuals with DP show typical susceptibility to the composite face illusion^18,42,43,61,62^, thought to be a key marker of holistic face processing^63,64^. Instead, we speculate that DPs have an apperceptive problem that affects local feature descriptions. Consistent with this possibility, many DPs are unable to accurately discriminate local face regions^2,32,60^. Similarly, variability in local feature encoding ability contributes substantially to the individual differences in face recognition seen in the typical population^65^.

It has recently been suggested that individual differences in face recognition ability may reflect different patterns of gaze fixations employed by observers. For example, some DPs appear to spend less time examining the internal feature of the face, in particular the eyes^66,67^. Conversely, so-called ‘super-recognizers’ – people with exceptional face recognition ability^68^ – spend more time inspecting the nose region than typical observers^66^. While these interesting differences warrant research attention in future, we believe it is unlikely that atypical gaze behaviour is driving the facial sex categorisation impairment seen here. In both tasks we employed a central fixation cross and brief stimulus presentation (250 ms) in order to limit variability in fixation behaviour as far as possible.

The principle line of evidence in favour of the mnemonic account of DP is the fact that some DPs perform worse on tests of face perception that have a memory component, than on tests that have little or no memory demand, notably the CFMT and CFPT, respectively^15,16,20^. It has recently been argued, however, that these apparent dissociations should be treated with caution^23^. In particular, these tasks are used differently in the diagnosis of DP. In many labs, a poor score on the CFMT is deemed necessary for a diagnosis of DP; i.e., people who describe face recognition problems outside the lab are excluded from DP research if their CFMT score does not fall at least two SDs below the typical mean. In contrast, the CFPT is treated as a descriptive measure, administered to assess differences in perceptual encoding ability. Thus, whereas DPs are preselected based on their low CFMT scores, their CFPT scores are free to vary. For example, only 9 out of 17 DPs in this sample performed more than two SDs below the normative mean on the CFPT. Given this treatment – akin to ‘double-dipping’ – it is not at all surprising that some DPs appear to perform worse on the CFMT than the CFPT^3^. It should also be noted that the psychometric properties of the CFPT are poorer than those of the CFMT, and that the simultaneous presentation of the faces in the CFPT may make it easier to employ compensatory strategies (e.g., detection of trivial pictorial cues).

We have argued that subtle deficits of facial sex categorisation may be widespread in DP, consistent with an apperceptive impairment. If this is the case, why do DPs rarely report problems making sex judgements in their daily lives^37,69^ ? We believe this apparent discrepancy reflects the different demands posed by sex categorisation outside of the lab and in contrived lab tasks. Outside of the lab, we rarely encounter people who seek to obscure their sex; on the contrary, individuals frequently elect to accentuate sexually dimorphic face signals through the use of make-up, personal grooming, and cosmetic surgery. Moreover, a wealth of non-face cues (e.g., facial hair, hair styles, clothes, use of make-up, body shape, voice) can be used to judge the sex of the faces we encounter outside the lab. Only when these cues are systematically removed in contrived lab-based studies, are individuals forced to base their sex categorisation judgements on facial structure. It is only under these conditions that DPs are disadvantaged relative to TD controls.

In summary, this study aimed to determine whether DPs exhibit deficits of facial sex discrimination. Our two complementary psychophysical procedures produced converging results: when judging closely controlled stimuli in which the strength of the sex signals were manipulated systematically, DPs were less able to categorise facial sex than matched TD controls. While these subtle deficits may have little impact on the day-to-day interactions of individuals with DP, they have considerable theoretical significance. Crucially, they imply that the condition has a locus of impairment early in the face processing stream, that hinders the ability of individuals to form structural descriptions of faces.

## Supplementary information

Supplementary Materials

Dataset 1

## Data Availability

The datasets generated during and/or analysed during the current study are available from the corresponding author on reasonable request.
