# Supplementary material for: The discrimination of facial sex in developmental prosopagnosia
Source: Sci Rep. 2019 Dec 13;9:19079. doi: 10.1038/s41598-019-55569-x (PMC6910918; doi:10.1038/s41598-019-55569-x)
Supplement: Supplementary file 1 — Supplementary Materials [file 41598_2019_55569_MOESM1_ESM.docx]

**The discrimination of facial sex in developmental prosopagnosia**

**Jade E. Marsh, Federica Biotti, Richard Cook, Katie L.H. Gray**

**Supplementary analyses**

**Sex discrimination task: Criterion analyses**

As described in the manuscript, *C* values were submitted to ANOVA with Difficulty (20%, 30%, 40%, 50%) as a within-participants factor and Group (TD, DP) as a between-participants factor. There was a significant main effect of Difficulty [*F*(1.76, 61.69) = 42.84, *p*  < .001, η_p_^2^ = .55]. There was no main effect of Group [*F*(1, 35) = 2.18, *p*  = .149, η_p_^2^ = .06], and no Group × Difficulty interaction [*F*(1.76, 61.69) = .24, *p*  = .760, η_p_^2^ = .01].

At the 20% (*M* = -.32, *SD* = .56; [*t*(36) = 3.48, *p* = 001]) and 30% (*M*= -.18, *SD* = .49; [*t*(36) = 2.16, *p* = .038]) morph-levels, participants were slightly biased to respond ‘female’. At the 40% morph-level (*M* = -.03, *SD* = .48), participants were unbiased in their responses [*t*(36) = .41, *p* = .687], whereas at the 50% morph-level (*M* = .18, *SD* = .39), participants were slightly biased to respond ‘male’ [*t*(36) = 2.78, *p* = .008].

Bias at each morph-level significantly differed from each other morph-level (20% morph-level compared with 30%: [*t*(36) = 5.15, *p* < .001], 40%: [*t*(36) = 5.57, *p* < .001], 50%: [*t*(36) = 8.22, *p* < .001]), 30% morph-level compared with 40%: [*t*(36) = 3.76, *p* = .001], 50%: [*t*(36) = 7.29, *p* < .001], 40% morph-level compared with 50%: *t*(36) = 5.85, *p* < .001]).


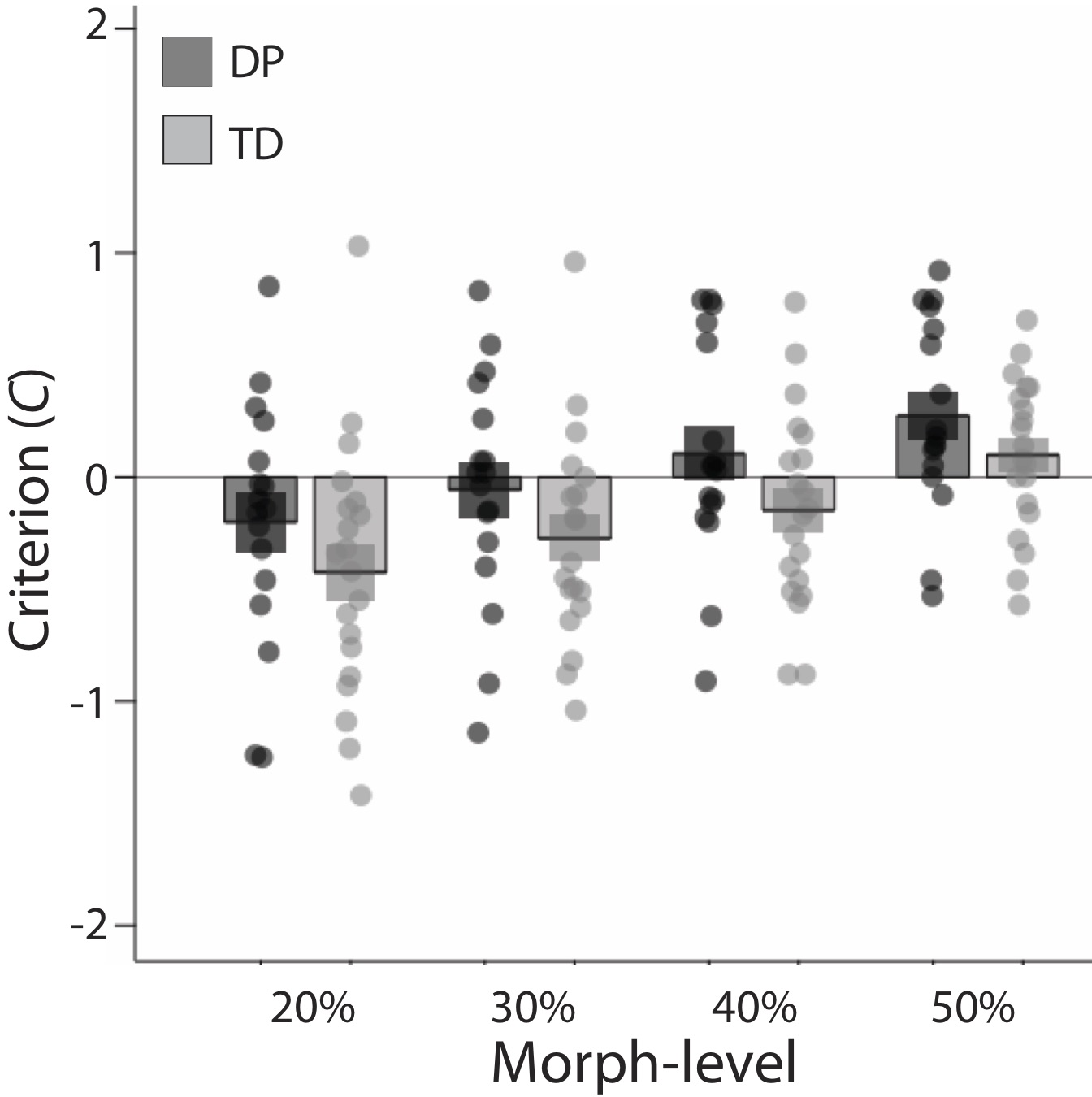


**Figure S1**. Criterion values for the sex discrimination task at each morph level and for each group. Note: bar height gives the mean, bands give the mean ±1 SEM.

**Table S1.**Z-scores for each developmental prosopagnosic (DP) on the morph categorisation task (slope) and sex discrimination task (*d’*).

| Participant | Slope z-score | *d'* 20% z-score | *d'* 30% z-score | *d'* 40% z-score | *d'* 50% z-score |
| --- | --- | --- | --- | --- | --- |
| F1 | 1.00 | 0.73 | 1.59 | -0.55 | -0.52 |
| F2 | -0.04 | -0.76 | 0.95 | 0.83 | 0.55 |
| F3 | -1.18 | -4.40 | -2.11 | -2.81 | -2.07 |
| F4 | -0.78 | -0.76 | 0.67 | -1.03 | -0.02 |
| F5 | -0.97 | -3.24 | -2.04 | -1.09 | -0.88 |
| F6 | -1.71 | -5.23 | -2.96 | -2.77 | -2.91 |
| F7 | -0.35 | -3.24 | -2.36 | -0.74 | -0.88 |
| F8 | -2.04 | -3.04 | -4.95 | -4.25 | -4.69 |
| F9 | 1.28 | 2.27 | 1.24 | 1.27 | 0.84 |
| F10 | 0.67 | -0.98 | -0.83 | 0.83 | -0.02 |
| M1 | -0.97 | -3.24 | -2.04 | -1.09 | -0.88 |
| M2 | -0.74 | -3.52 | -1.82 | 0.93 | -0.56 |
| M3 | -1.31 | -5.61 | -4.71 | -2.39 | -3.58 |
| M4 | -0.69 | -0.32 | -1.47 | -1.45 | -0.88 |
| M5 | -0.09 | -3.08 | -2.82 | -1.95 | -0.96 |
| M6 | 0.57 | -1.92 | -0.86 | -0.17 | -0.94 |
| M7 | -1.32 | -4.57 | -3.28 | -2.37 | -2.30 |
| DP Mean | 6.00 | 1.19 | 1.84 | 2.48 | 2.84 |
| DP SD | 2.75 | 0.42 | 0.54 | 0.74 | 0.70 |
| Control Mean | 7.97 | 1.66 | 2.30 | 3.01 | 3.42 |
| Control SD | 3.14 | 0.18 | 0.28 | 0.48 | 0.48 |

Note: To calculate z-scores, DPs’ slope and *d’* measures at each difficulty level were compared to the control sample for each task.
